# Nomogram model for predicting the long-term prognosis of cervical cancer patients: a population-based study in Mato Grosso, Brazil

**DOI:** 10.1186/s12885-025-14056-5

**Published:** 2025-04-14

**Authors:** Sancho Pedro Xavier, Noemi Dreyer Galvão, Marco Aurélio Bertúlio das Neves, Kátia Moreira da Silva, Adila de Queiroz Neves Almeida, Ageo Mario Cândido da Silva

**Affiliations:** 1 Present Address: Federal University of Mato Grosso - Institute of Collective Health, Av. Fernando Correa da Costa, nº 2367 - Bairro Boa Esperança. Cuiabá - MT, Cuiaba, MT 78060-900 Brazil; 2 State Secretary of Health of Mato Grosso, Cuiabá, Mato Grosso Brazil

**Keywords:** Cervical cancer, Nomogram, Overall survival, Prognostic factors, Mato Grosso

## Abstract

**Background:**

Cervical cancer (CC) is the third most common cancer among women worldwide and the second most prevalent neoplasm in Mato Grosso, Brazil, in 2020. This study aimed to analyze overall survival (OS), identify prognostic factors, and develop a nomogram to predict the long-term prognosis of CC patients using population-based data from Mato Grosso, Brazil.

**Methods:**

Integrated data from the Mortality Information System (SIM) and the Population-Based Cancer Registry (RCBP) were used for patients diagnosed with CC between 2001 and 2018. Group differences were analyzed using the Log-rank test, and survival analysis was performed using the Kaplan-Meier method. Univariable and multivariable Cox regression models were applied to identify predictors of OS. A nomogram was developed to predict OS at 1, 3, 5, and 10 years. The accuracy of the model was assessed using the C-index, receiver operating characteristic (ROC) curve, and calibration plots.

**Results:**

The median follow-up time was 12 years (range: 6.28 − 17.1). The OS rates at 1, 3, 5, and 10 years were 95.4%, 91.3%, 89.9%, and 88.3%, respectively. Age, histological type, and disease stage were identified as independent prognostic factors for OS. The C-index for OS was 0.869, and the areas under the ROC curve for 1, 3, 5, and 10 years were 0.910, 0.897, 0.895, and 0.884, respectively, indicating good discrimination. The nomogram demonstrated good agreement with the observed survival rates.

**Conclusion:**

The developed nomogram predicts OS for CC patients at 1, 3, 5, and 10 years, showing good concordance with the observed survival rates and serving as a useful tool for guiding personalized interventions. Notably, disease staging and histopathological type were the most significant prognostic factors for OS.

**Supplementary Information:**

The online version contains supplementary material available at 10.1186/s12885-025-14056-5.

## Introduction

Cervical cancer (CC) is the fourth most frequently diagnosed cancer among women of all ages worldwide [1] and the second most common among women of reproductive age [2]. It represents a significant public health challenge both globally and nationally, ranking among the leading causes of cancer-related deaths [3]. CC accounts for approximately 7.5% of all cancer deaths among women, with the highest incidence observed in the 35 to 65 age group [4]. The magnitude and distribution of the disease vary significantly across countries, influenced by factors such as access to healthcare services and the availability of early detection programs [5]. In Brazil, this is a pressing concern, as many women are still diagnosed at advanced stages of the disease [3, 6].

In 2020, approximately 16,710 new cases of CC were reported in Brazil, with an incidence rate of 16.35 per 100,000 women and a mortality rate of 5.33 per 100,000 women [7]. In the state of Mato Grosso, CC was the second most common neoplasm among women that year, with an incidence rate of 12.43 cases per 100,000 women [7]. Early detection plays a crucial role in patient survival, offering survival rates of up to 90%. However, Mato Grosso faces unique challenges in early diagnosis due to its geographical diversity [3]. A population-based study conducted in the state reported a 5-year cause-specific survival rate of 90% [8]. The prognosis of CC generally depends on several factors, including disease stage, histological type, and patient age [3, 6, 9]. Exploring these factors is essential to provide better guidance to clinical decision-makers through the development of prognostic models for personalized predictions [10].

Nomograms are statistical models used to calculate the probability of individual clinical events based on prognostic characteristics and determinants [10–14]. They transform complex regression equations into visual representations, simplifying result interpretation and clinical assessment [15–17]. These models have been applied to predict survival probabilities in patients with various cancer types through a specific scoring system [14, 15]. In Mato Grosso, no studies have yet utilized a population-based cancer registry covering the entire state or applied advanced statistical models, such as nomograms, to predict individual survival rates based on specific characteristics of diagnosed patients. This study aimed to analyze overall survival, identify prognostic factors, and develop a nomogram model to predict the long-term prognosis of CC patients diagnosed between 2001 and 2018, using population-based data from Mato Grosso, Brazil.

## Methods

### Study design, data collection, and setting

A retrospective cohort study was conducted using integrated data from the Population-Based Cancer Registry (RCBP) and the Mortality Information System (SIM). Data integration was achieved through deterministic linkage [8, 18], matching the patient’s name, mother’s name, and date of birth to ensure accurate matching. Cancer incidence data were obtained from the Mato Grosso Population-Based Cancer Registry, covering the period from 2001 to 2018. Mortality data, spanning from 2000 to 2022, represent the most current records available. Both datasets were sourced from the Mato Grosso State Health Department (SES-MT).

The state of Mato Grosso is located in the Central-West region of Brazil and is the third largest in area, with approximately 3,658,649 inhabitants distributed across 142 municipalities and 16 health regions [19, 20]. Most of the population resides in the capital (Cuiabá) [19]. Oncology care is provided by five high-complexity centers, three in the capital and two in the interior (Sinop and Rondonópolis) [21]. The state’s economy is driven by agribusiness and has the highest pesticide consumption rate in the country [22].

### Population and sample size

The study population included all patients diagnosed with CC (topography code C53) based on the International Classification of Diseases for Oncology, Third Edition (ICD-O-3) [23], between 2001 and 2018. Observations with missing data or unknown disease stage were excluded. A total of 1,682 patients were included in the analysis.

### Variables

The variables analyzed in this study included both sociodemographic and clinical factors of the patients. Sociodemographic variables encompassed age (≤ 40 years, 41–50 years, 51–65 years, > 65 years), race (White, Black, other/unknown), educational level (primary, secondary, higher education, no education, unknown), city of residence (Cuiabá, Várzea Grande, Rondonópolis, Sinop, and others), year of diagnosis, survival time (in years), follow-up duration, and vital status. Clinical variables included the histopathological type of cancer (e.g., squamous cell carcinoma, adenocarcinoma, malignant neoplasms, and others) and stage of cancer at diagnosis, categorized as in situ, localized, or metastatic.

The outcome variable was the time to death for CC patients, measured in years from the date of diagnosis. Death was coded as an event (1), while patients who did not die during the follow-up period were censored (0).

### Statistical analysis

Descriptive analyses were conducted for categorical variables, presenting absolute and relative frequencies. For quantitative variables, the Shapiro-Wilk test was applied to assess data normality, with results expressed as medians and interquartile ranges (IQRs) of 25% and 75%. Survival models were employed to analyze the time-to-event occurrence, using non-parametric approaches to handle censored data. Survival estimates were calculated using the Kaplan-Meier method, and survival differences between groups were evaluated with the Log-rank test [24]. Additionally, the Tarone-Ware and Peto-Prentice tests were applied to examine differences among survival curves [25].

The Cox proportional hazards regression models, both univariable (CHR) and multivariable (AHR), were used to evaluate the impact of prognostic factors on patient survival times. Variables with statistically significant p-values from the Log-rank, Tarone-Ware, or Peto-Prentice tests were included in the models. Hazard ratios (HR) and 95% confidence intervals (CI) were calculated. To finalize the model, the Akaike Information Criterion (AIC) was utilized, where lower AIC values indicated a better trade-off between model fit and complexity [3]. The proportional hazards assumption was assessed using the goodness-of-fit (GOF) test with Schoenfeld residuals (details provided in the supplements). A p-value < 0.05 was considered statistically significant. Based on the results of the multivariable Cox analysis, statistically significant variables were included in the nomogram model to predict individualized survival probabilities.

The nomogram was constructed by proportionally converting the regression coefficients of each independent risk factor from the multivariable Cox model into a score ranging from 0 to 100 points [26]. The total score for each patient was obtained by summing the points assigned to each variable, which was then used to estimate the predicted probability of survival. The model’s performance was evaluated using the concordance statistic (C-index), the area under the receiver operating characteristic curve (AUC), and calibration. The C-index is equivalent to the AUC of the ROC curve, with a value of 0.5 indicating no predictive discrimination and a value of 1.0 indicating perfect prediction of outcomes. Calibration was assessed using 1,000 bootstrap samples to minimize overfitting, and the results were visualized with a calibration plot. In a well-calibrated model, the prediction curve aligns closely with the 45-degree diagonal line. Additionally, the model’s accuracy was confirmed by analyzing the ROC curve.

Data analysis was performed using R software version 4.4.1 (R Project for Statistical Computing, RRID: SCR_001905) and RStudio version 2024.09.1 + 394, released on 2024-11-04.

## Results

### Sociodemographic and clinical characteristics of the patients

Initially, the database included 2,284 patients diagnosed with CC between 2001 and 2018. After applying the eligibility criteria, 602 patients were excluded due to missing data (*n* = 30) or unknown disease stage (*n* = 572), resulting in a final sample of 1,682 patients. The sociodemographic and clinical characteristics of these patients are summarized in Table 1. The majority (55.9%) were 40 years old or younger, followed by the age groups 41–50 years (19.9%) and 51–65 years (16.6%). Only 7.5% of the patients were 65 years or older. Regarding histopathological characteristics, squamous cell carcinoma was the most frequent type, accounting for 92.7% of cases, followed by adenocarcinoma (3.4%) and others (2.3%). As for cancer staging, most patients (76.5%) were diagnosed at the in situ stage, followed by localized stage (18.7%) and metastatic stage (4.8%).

**Table 1:** Characteristics of eligible patients diagnosed with CC and analysis of factors associated with survival

		Survival status		*p*-valor		
Predictors	Total *n* = 1682	Alive *n* = 1482	Died *n* = 200	Log-rank	Tarone-Ware	Gehan-Breslow
**Age**						
≤ 40	941 (55.9)	906 (96.3)	24 (3.7)			
41–50	335 (19.9)	283 (84.5)	52 (15.5)	*p* < 0.001	*p* < 0.001	*p* < 0.001
51–65	280 (16.6)	218 (77.9)	62 (22.1)			
> 65	126 (7.5)	75 (59.5)	51 (40.5)			
**Skin color**						
White	437 (26.0)	370 (84.7)	67 (15.3)			
Black	91 (5.4)	79 (86.8)	12 (13.2)	*p* = 0.02	*p* < 0.001	*p* < 0.001
Other/Unknown	1154 (68.6)	1033 (89.5)	121 (10.5)			
**Educational level**						
Elementary	100 (5.9)	45 (45.0)	55 (55.0)			
Middle school	43 (2.6)	29 (67.4)	14 (32.6)			
No formal education	21 (1.2)	8 (38.1)	13 (61.9)	*p* < 0.001	*p* = 0.028	*p* = 0.030
High education	14 (0.8)	8 (57.1)	6 (42.9)			
No information	1504 (89.4)	1392 (92.6)	112 (7.4)			
**City of residence**						
Cuiaba	426 (25.3)	362 (85.0)	64 (15.0)			
Varzea Grande	162 (9.6)	146 (90.1)	16 (9.9)			
Rondonopolis	112 (6.7)	89 (79.5)	23 (20.5)	*p* = 0.002	*p* = 0.003	*p* = 0.005
Sinop	42 (2.5)	39 (92.9)	3 (7.1)			
Other	940 (55.9)	846 (90.0)	94 (10.0)			
**Histopathology type**						
Squamous cell carcinoma	1559 (92.7)	1403 (90.0)	156 (10.0)			
Adenocarcinoma	57 (3.4)	43 (75.4)	14 (24.6)			
Malignant neoplasms	27 (1.6)	7 (25.9)	20 (74.1)	*p* < 0.001	*p* < 0.001	*p* < 0.001
Others	39 (2.3)	29 (74.4)	10 (25.6)			
**Stage**						
In situ	1286 (76.5)	1240 (96.4)	46 (3.6)			
Localized	315 (18.7)	206 (65.4)	109 (34.6)	*p* < 0.001	*p* < 0.001	*p* < 0.001
Metastatic	81 (4.8)	36 (44.4)	45 (55.6)			
**Year of diagnosis**						
2001–2005	608 (36.1)	512 (84.2)	96 (15.8)			
2006–2010	415 (24.7)	377 (90.8)	38 (9.2)	*p* = 0.001	*p* = 0.001	*p* < 0.001
2011–2015	394 (23.4)	345 (87.6)	49 (12.4)			
2016–2018	265 (15.8)	248 (93.6)	17 (6.4)			

### Kaplan-Meier survival estimates and analysis of factors associated with patient survival

**Fig. 1:**
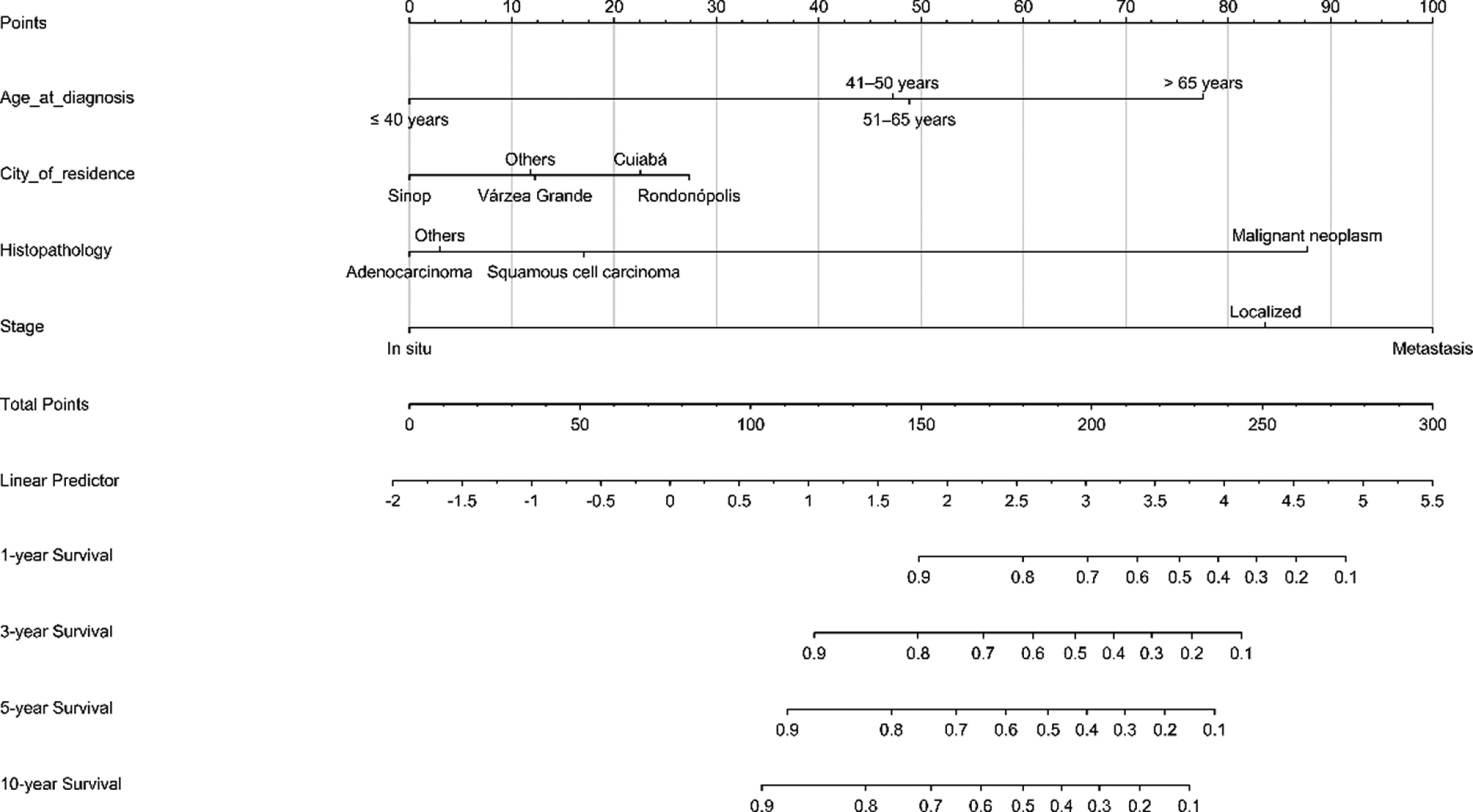
Nomogram model for predicting 1-, 3-, 5-, and 10-year survival rates in CC patients

The median follow-up time for patients was 12 years (interquartile range: 6.28 − 17.13 years), with a maximum follow-up of 21 years. The overall survival (OS) rate at the end of 21 years was 87.2% (95% CI: 85.5–88.9), while at the end of 12 years, it was 87.9% (95% CI: 86.3–89.5). Among all patients, OS rates were 95.4% (95% CI: 94.4–96.4) at 1 year, 91.3% (95% CI: 90.0–92.7) at 3 years, 89.9% (95% CI: 88.4–91.3) at 5 years, and 88.3% (95% CI: 86.7–89.9) at 10 years. The corresponding survival rates and curve are shown in Supplementary Materials (S1): Fig. 1; Table 1.

Kaplan-Meier survival estimates at 1, 3, 5, and 10 years indicated statistically significant differences across the compared groups. Regarding age, patients aged 40 years or younger exhibited the highest survival rates at all analyzed periods: 1 year (99%), 3 years (98%), 5 years (97%), and 10 years (96%). In contrast, those over 65 years old had the lowest survival rates: 1 year (76%), 3 years (67%), 5 years (63%), and 10 years (59%). In terms of histopathology type, patients with squamous cell carcinoma demonstrated the highest survival rates over time: 1 year (97%), 3 years (93%), 5 years (92%), and 10 years (90%). Conversely, patients with malignant neoplasms had significantly lower survival rates: 30% at 1 year, 26% at 3, 5, and 10 years, respectively. Patients with metastatic stage showed the poorest survival outcomes: 1 year (80%), 3 years (51%), 5 years (48%), and 10 years (44%), as detailed in Table 2.

The analysis of factors associated with survival, presented in Table 1, revealed significant differences in survival rates based on various demographic and clinical characteristics of the patients.

**Table 2:** Kaplan-Meier estimated survival rates at 1, 3, 5, and 10-years among CC patients

	Survival Rate (95% CI)			
Predictors	1-Year	3-Year	5-Year	10-Year
**Age**				
≤ 40	99 (99; 100)	98 (97; 99)	97 (96; 98)	96 (95; 98)
41–50	96 (93; 98)	90 (86; 93)	87 (83; 91)	85 (81; 89)
51–65	91 (88; 94)	84 (79; 88)	81 (77; 86)	79 (74; 84)
> 65	76 (69; 84)	67 (59; 75)	63 (55; 72)	59 (51; 68)
**Skin color**				
White	93 (91; 96)	89 (86; 92)	87 (84; 90)	85 (82; 89)
Black	99 (97; 100)	90 (84; 96)	86 (83; 88)	83 (81; 86)
Other/Unknown	96 (95; 97)	92 (91; 94)	91 (90; 93)	90 (88; 92)
**Educational level**				
Elementary	84 (77; 92)	63 (54; 73)	57 (48; 68)	48 (39; 59)
Middle school	91 (82; 100)	79 (68; 92)	77 (65; 90)	72 (60; 87)
No formal education	81 (66; 100)	71 (54; 94)	52 (35; 79)	43 (26; 70)
High education	93 (80; 100)	57 (36; 90)	57 (36; 90)	57 (36; 90)
No information	96 (96; 97)	94 (93; 95)	93 (92; 95)	93 (91; 94)
**City of residence**				
Cuiabá	95 (93; 97)	90 (87; 93)	88 (85; 91)	84 (81; 88)
Várzea Grande	97 (94; 100)	93 (89; 97)	91 (86; 95)	91 (86; 95)
Rondonópolis	90 (85; 96)	86 (79; 92)	84 (77; 91)	79 (72; 87)
Sinop	95 (89; 100)	93 (85; 100)	93 (85; 100)	93 (85; 100)
Other	96 (95; 97)	92 (91; 94)	91 (89; 93)	91 (89; 92)
**Histopathology type**				
Adenocarcinoma	89 (82; 98)	81 (71; 92)	75 (65; 87)	75 (65; 87)
Squamous cell carcinoma	97 (96; 98)	93 (92; 94)	92 (90; 93)	90 (89; 92)
Malignant neoplasms	30 (17; 53)	26 (14; 49)	26 (14; 49)	26 (14; 49)
Other	85 (74; 97)	79 (68; 93)	77 (65; 91)	97 (65; 91)
**Stage**				
In Situ	99 (99; 100)	98 (97; 99)	97 (97; 98)	96 (95; 98)
Localized	84 (80; 88)	75 (70; 80)	70 (65; 75)	67 (61; 72)
Metastatic	80 (72; 89)	51 (41; 63)	48 (38; 60)	44 (34; 57)
**Year of diagnosis**				
2001–2005	93 (91; 95)	89 (86; 91)	87 (84; 89)	85 (83; 88)
2006–2010	98 (96; 99)	95 (93; 97)	94 (92; 96)	91 (89; 94)
2011–2015	96 (94; 98)	90 (87; 93)	88 (85; 92)	87 (84; 91)
2016–2018	97 (94; 99)	94 (91; 97)	94 (91; 97)	-------------

Note: Others, sarcoma, carcinosarcoma, carcinosarcoma, carcinoma not otherwise specified (NOS), undifferentiated carcinoma; CI, Confidence Interval. The 10-year survival estimate was not calculated for the 2016–2018 cohort due to insufficient follow-up time (data available only until 2022)

### Cox proportional hazards analysis for independent predictors of OS

Cox regression analyses were performed to identify prognostic factors associated with OS, as detailed in Table 3. In the univariable analysis, all variables showed a significant association with OS. Multicollinearity among the predictor variables was assessed, and no significant issues were identified (see S1. Table 2; Fig. 3). In the final model (Model 2), the variables associated with OS were: age (classified into the following groups: 41–50, 51–65, and > 65 years), histopathology type (malignant neoplasms), and disease stage (localized and metastatic).

**Table 3:** Univariable and multivariable Cox regression for OS

	Univariable Model		Multivariable Model			
			Model 1		Model 2	
Predictors	CHR (95% CI)	*p*-value	AHR (95% CI)	*p*-value	AHR (95% CI)	*p*-value
**Age**						
≤ 40	Reference		Reference		Reference	
41–50	4.39 (2.86; 6.73)	*p* < 0.001	3.10 (1.99; 4.81)	*p* < 0.001	3.20 (2.07; 4.94)	*p* < 0.001
51–65	6.57 (4.34; 9.95)	*p* < 0.001	3.40 (2.29; 5.27)	*p* < 0.001	3.29 (2.14; 5.05)	*p* < 0.001
> 65	14.38 (9.35; 22.12)	*p* < 0.001	6.26 (3.97; 9.88)	*p* < 0.001	7.00 (4.46; 11.01)	*p* < 0.001
**Skin color**						
White	Reference		Reference		Reference	
Black	0.84 (0.45; 1.54)	*p* = 0.565	0.53 (0.28; 1.02)	*p* = 0.057	0.58 (0.31; 1.10)	*p* = 0.097
Other/Unknown	0.66 (0.49; 0.89)	*p* = 0.007	0.81 (0.59; 1.13)	*p* = 0.212	0.88 (0.64;1.20)	*p* = 0.413
**City of residence**						
Other	Reference		Reference		Reference	
Cuiabá	1.54 (1.12; 2.11)	*p* = 0.008	1.30 (0.94; 1.80)	*p* = 0.115	1.30 (0.94; 1.80)	*p* = 0.106
Várzea Grande	0.99 (0.58; 1.70)	*p* = 0.999	1.06 (0.62; 1.82)	*p* = 0.833	1.07 (0.62; 1.83)	*p* = 0.818
Rondonópolis	2.17 (1.37; 3.42)	*p* < 0.001	1.48 (0.93; 2.36)	*p* = 0.100	1.47 (0.92; 2.34)	*p* = 0.104
Sinop	0.71 (0.22; 2.24)	*p* = 0.557	0.74 (0.23; 2.36)	*p* = 0.610	0.76 (0.24; 2.42)	*p* = 0.642
**Histopathology type**						
Squamous cell carcinoma	Reference		Reference		Reference	
Adenocarcinoma	2.72 (1.58; 4.71)	*p* < 0.001	0.64 (0.36; 1.12)	*p* = 0.117	0.63 (0.36; 1.11)	*p* = 0.101
Malignant neoplasms	22.89 (14.31; 36.60)	*p* < 0.001	5.86 (3.41; 10.09)	*p* < 0.001	5.61 (3.29; 9.55)	*p* < 0.001
Others	2.84 (1.50; 5.38)	*p* < 0.001	0.72 (0.38; 1.39)	*p* = 0.333	0.69 (0.36; 1.33)	*p* = 0.268
**Stage**						
In situ	Reference		Reference		Reference	
Localized	11.58 (8.20; 16.34)	*p* < 0.001	8.46 (5.72; 12.52)	*p* < 0.001	7.81 (5.40; 11.29)	*p* < 0.001
Metastatic	22.89 (15.15; 34.60)	*p* < 0.001	12.21 (7.59; 19.66)	*p* < 0.001	12.36 (7.74; 19.74)	*p* < 0.001
**Year of diagnosis**						
2016–2018	Reference		Reference			
2001–2005	2.26 (1.34; 3.79)	*p* = 0.002	1.12 (0.62; 2.01)	*p* = 0.703	-----------------------	------------
2006–2010	1.25 (0.71; 2.23)	*p* = 0.442	1.56 (0.62; 2.87)	*p* = 0.150	-----------------------	------------
2011–2015	1.83 (1.05; 3.19)	*p* = 0.032	1.49 (0.83; 2.68)	*p* = 0.182	-----------------------	------------
**AIC**			2521.99		2520.48	

### Development of the nomogram prognostic model

The prognostic factors identified in multivariable model 2 were used to develop the nomogram, designed to estimate OS probabilities at 1, 3, 5, and 10 years (Fig. 1). Each prognostic parameter was assigned a score reflecting its predictive value, and the cumulative score was then used to estimate OS probabilities at these specified time points. The total score of the variables was subsequently converted into an estimated probability of death. For example, consider a patient aged 51 to 65 years, diagnosed with squamous cell carcinoma of the cervix at a localized stage, and residing in Cuiabá. The assigned scores for these characteristics were as follows: age 51–65 years (49 points), squamous cell carcinoma (17 points), localized stage (84 points), and residence in Cuiabá (23 points). The total score for this patient would be 173, yielding estimated survival probabilities of approximately 80%, 70%, 60%, and 50% for 1, 3, 5, and 10 years, respectively.

### Nomogram validation

**Fig. 2:**
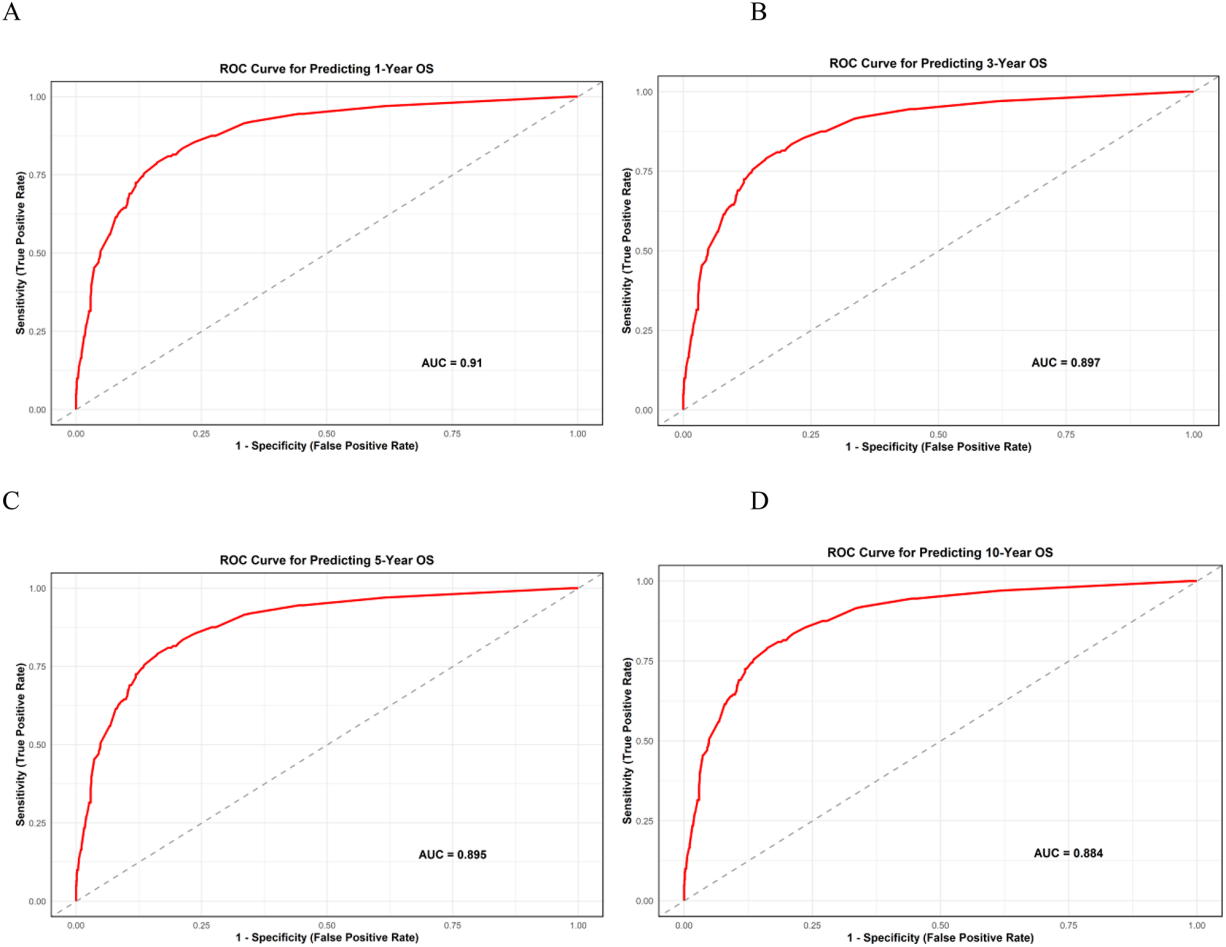
ROC Curves for 1-, 3-, 5-, and 10-Year OS Predictions from the Nomogram Model. The ROC curve illustrates survival predictions for patients at (A) 1 year, (B) 3 years, (C) 5 years, and (D) 10 years. The false positive rate (FPR) is shown on the X-axis, and the true positive rate (TPR) is shown on the Y-axis

**Fig. 3:**
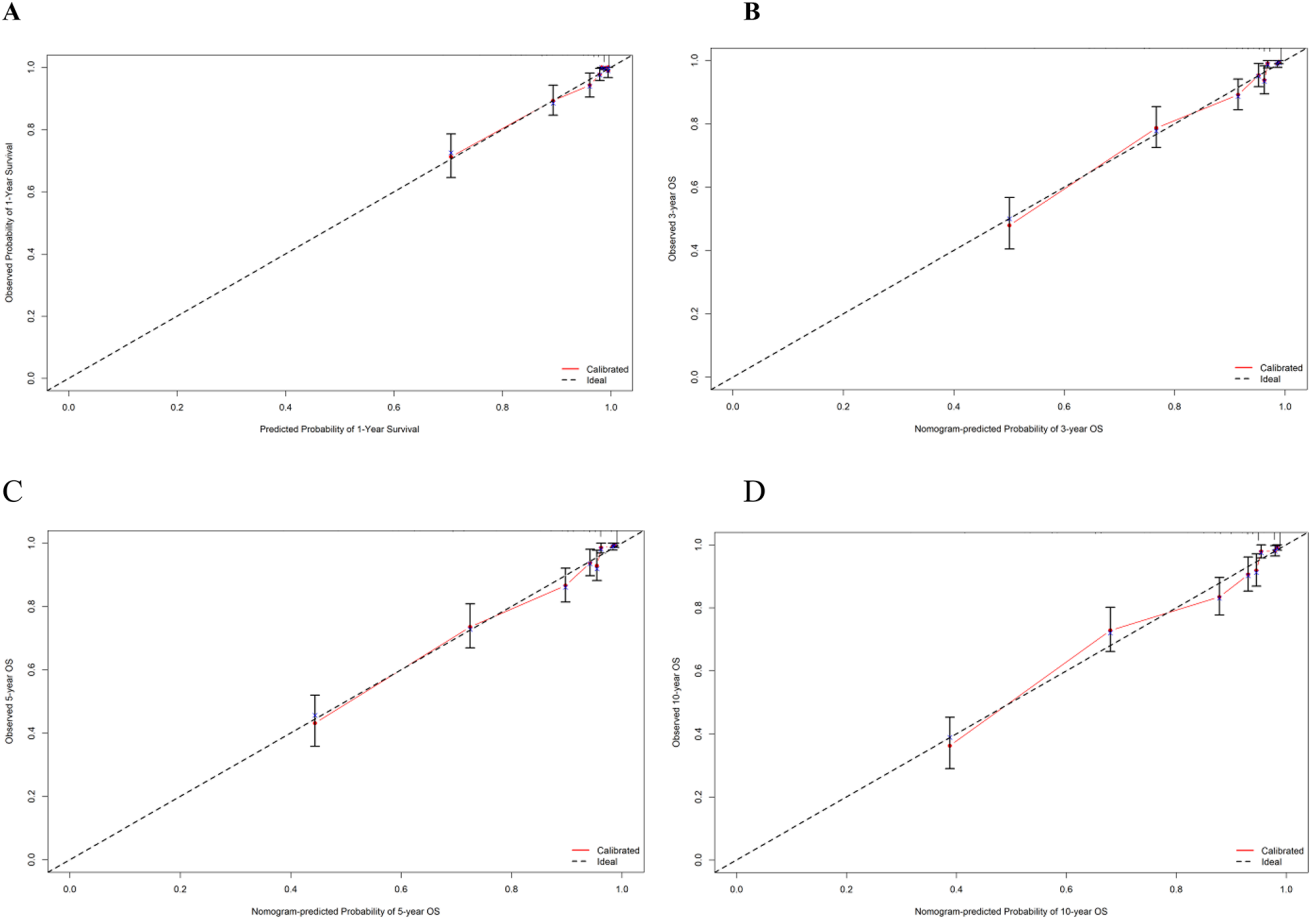
Calibration plots for the nomogram model, illustrating the comparison between predicted probabilities and actual observed probabilities for 1-, 3-, 5-, and 10-year OS in patients with CC (A: 1 year, B: 3 years, C: 5 years, D: 10 years)

The predictive performance of the nomogram was evaluated using multiple methods. First, the model’s discriminatory ability was assessed with the C-index and the AUC. The nomogram’s C-index for OS was 0.869, demonstrating satisfactory discrimination. The AUCs for predicting OS at 1, 3, 5, and 10 years were 0.910, 0.897, 0.895, and 0.884, respectively, with an average AUC of 0.883 across all time points, highlighting the strong discriminatory capability of the nomogram (S1. Figure 2, and Fig. 2). Next, the model’s calibration was evaluated using calibration curves (Fig. 3), which illustrated the agreement between the nomogram’s predictions and the observed survival outcomes. The calibration curves demonstrated good agreement between the predicted probabilities and the observed outcomes, emphasizing the accuracy of the nomogram’s calibration performance.

## Discussion

In the present study, the OS rate of patients diagnosed with CC was 87.2%, with OS rates of 95.4%, 91.3%, 89.9%, and 88.3% at 1, 3, 5, and 10 years, respectively. By comparison, recent studies have reported varied outcomes: in Malaysia, OS rates were 97%, 79%, and 71% at 1-, 3-, and 5-year, respectively [27]; in China, the rates were 91.0% at 1-year and 84.4% at 5-year [28]; in the United States, OS rates were 74.4% at 3 years and 67.7% at 5 years [29]; and in northeastern Thailand, survival rates were 77.4%, 49.4%, and 43.2% at 1, 5, and 10 years, respectively [30]. Additionally, a meta-analysis of studies conducted in Asian countries reported survival rates of 76.6% at 1 year, 68.8% at 3 years, 62.3% at 5 years, and 61.6% at 10 years [31]. These regional variations in survival rates may reflect differences in healthcare access, early diagnosis practices, therapeutic approaches, and population-specific characteristics. The literature emphasizes that survival rates for patients with CC are strongly influenced by the stage at diagnosis, with the disease stage being a crucial determinant of prognosis [31]. In the present study, survival rates showed minimal differences across the 1-, 3-, 5-, and 10-year time points. Despite Mato Grosso’s heterogeneous socioeconomic contexts and epidemiological indicators [20], this finding may suggest improvements in early screening and diagnosis, enabling detection at earlier stages and, consequently, better treatment outcomes.

Multivariable Cox models were used to examine factors influencing the prognosis of patients with CC. The independent predictors associated with a poorer prognosis included age at diagnosis, histopathology type, and disease stage. The results demonstrated that increasing age was linked to reduced survival, consistent with findings from previous studies [3, 29, 32]. This association can be explained by the fact that older patients tend to have more comorbidities, which can impact disease management and limit available therapeutic options. As such, older patients typically receive less aggressive treatments compared to younger patients [3]. Another possible explanation is that older patients are often diagnosed at later stages of the disease, which may worsen the prognosis. This delayed diagnosis can be influenced by structural barriers within the healthcare system, such as fragmented access to specialized care, insufficient screening coverage, and deficiencies in early detection [3]. Socioeconomic factors may also play a role, as lower-income populations often face difficulties in accessing timely diagnosis and treatment, exacerbating disparities in cancer outcomes [33].

Interestingly, our findings indicate that patients diagnosed with CC of the malignant neoplasm type have a higher risk of poorer prognosis compared to those with squamous cell carcinoma (SCC), a result consistent with previous studies [9, 30]. However, some studies have reported different associations, suggesting that patients with adenocarcinoma (ADC) type CC have a worse survival prognosis compared to those with SCC [17, 34], although the results were inconsistent across studies [29, 32, 35]. Several factors may explain these discrepancies. First, population composition differs between studies; while our study included patients from all disease stages, others have focused on specific cases, such as those with metastases or stage III C1 disease [9, 34]. Additionally, the lower prevalence of ADC reduces the sample size for this histopathology type, potentially limiting statistical power and significance [36]. Nonetheless, evidence suggests that patients with malignant neoplasms or ADC-type CC generally experience a poorer prognosis compared to those with SCC. This may be due to the more aggressive biological behavior, late detection, and a greater likelihood of distant recurrence linked to genetic factors. Since SCC originates from ectocervical cells, it is more readily detected through screening programs like the Pap smear. In contrast, ADC arises from glandular cells in the endocervix, often going undetected until more advanced stages [37]. Furthermore, our results showed that patients with localized or metastatic stage had a higher risk of death compared to those diagnosed at the in situ stage, a finding supported by several previously published studies [15, 28, 29, 32, 38]. This increased risk may be due to the cancer becoming more aggressive and difficult to treat as it spreads or invades tissues beyond its original site. In contrast, the in situ stage is generally more treatable and associated with a better prognosis. The disparities in detection between these histological types further highlight the role of screening programs and access to healthcare in shaping CC outcomes.

Finally, a nomogram was developed using the predictor variables from the multivariable model 2 to estimate OS probabilities at 1, 3, 5, and 10 years for patients diagnosed with CC. Recent studies have created predictive nomograms to estimate the prognosis of CC patients, aiding healthcare professionals in planning personalized interventions [29, 32, 34, 35]. These models, built using large population-based databases such as SEER, incorporate a wide range of prognostic variables, including TNM staging, treatment modalities, and tumor classification according to the International Federation of Gynecology and Obstetrics (FIGO), among others [39, 40]. However, despite the extensive datasets these sources provide, they do not always accurately reflect specific regional realities, particularly in settings with distinct or less structured healthcare systems. Moreover, considering that genetic variations across different regions of the world significantly influence disease prognosis, the absence of Brazilian patients in these analyses represents a relevant limitation, particularly for those residing in the state of Mato Grosso.

The nomogram model developed in this study identified cancer stage at diagnosis as a major factor influencing predicted OS probabilities at 1, 3, 5, and 10 years, a finding consistent with other research [15, 17, 29, 32, 41, 42]. Unlike prior studies that used the FIGO staging system, our study categorized cancer into three main stages (in situ, localized, and metastatic) based on the extent of disease spread. Additionally, while some studies [17, 32, 34] did not emphasize histopathological type as a key prognostic factor, our findings identified it as the second most significant predictor, aligning with results from another study [29]. The model’s performance was evaluated using the C-index, AUC, and calibration curves. In this study, the C-index was 0.869, indicating satisfactory discriminatory ability. In comparison, previous studies have reported nomogram C-indices ranging from 0.65 to 0.831 [28, 32, 34, 35]. The AUC values for predicting OS at 1, 3, 5, and 10 years were 0.910, 0.897, 0.895, and 0.884, respectively, reflecting high model accuracy and outperforming those reported in other studies [9, 15, 17, 28]. Moreover, the calibration curves demonstrated that the predicted survival rates at these time points closely matched the actual observed outcomes, indicating that the model provided reliable and accurate survival predictions.

This study has several limitations. First, as a retrospective analysis, the data were sourced from two integrated databases. During the data merging process, patients with missing information for key variables, such as unknown staging and age, were excluded, potentially introducing selection bias. Second, certain critical clinical and non-clinical variables were not available in the database records, including marital status at diagnosis, HPV status, HPV vaccination history, FIGO stage, tumor size, treatment modalities (chemotherapy, surgery, and radiotherapy), and the number of metastases. Future studies should aim to integrate these variables to enhance model accuracy and applicability. Additionally, as the data were sourced from secondary databases, the underreporting of deaths cannot be ruled out, particularly for patients outside the monitored system. Lastly, the nomogram underwent only internal validation and has not yet been externally validated, underscoring the need for future studies to enhance its accuracy and expand its applicability. To strengthen confidence in the model, future research should include external validation using an independent dataset or another Brazilian cancer registry. Despite these limitations, this study represents a pioneering effort in Brazil, particularly in Mato Grosso. It leverages a robust, population-based cancer registry encompassing all 141 municipalities over an 18-year period. This comprehensive dataset offers valuable insights to inform personalized interventions, shape public health policies, and improve CC patient outcomes. Additionally, the nomogram provides an individualized assessment tool, aiding clinical decision-making through targeted interventions and paving the way for further research into innovative diagnostic and treatment approaches.

## Conclusion

The prognostic factors identified for overall survival were age, histopathology type, and cancer staging at the time of diagnosis. Based on the results from the multivariable Cox proportional hazards model, a nomogram was developed, which showed good concordance with the actual survival rates, proving to be a useful tool for guiding personalized interventions. Notably, staging, histopathology type, and age at diagnosis were the main prognostic factors for OS.

## Electronic supplementary material

Below is the link to the electronic supplementary material.

Supplementary Material 1

## Data Availability

The datasets used and/or analyzed during the current study are available from the corresponding author upon reasonable request.
