# Supplementary material for: Nomogram model for predicting the long-term prognosis of cervical cancer patients: a population-based study in Mato Grosso, Brazil
Source: BMC Cancer. 2025 Apr 14;25:684. doi: 10.1186/s12885-025-14056-5 (PMC11995657; doi:10.1186/s12885-025-14056-5)
Supplement: Supplementary file 1 — Supplementary Material 1 [file 12885_2025_14056_MOESM1_ESM.docx]

**Supplementary Materials (S1)**

**S1. Figure 1**

**
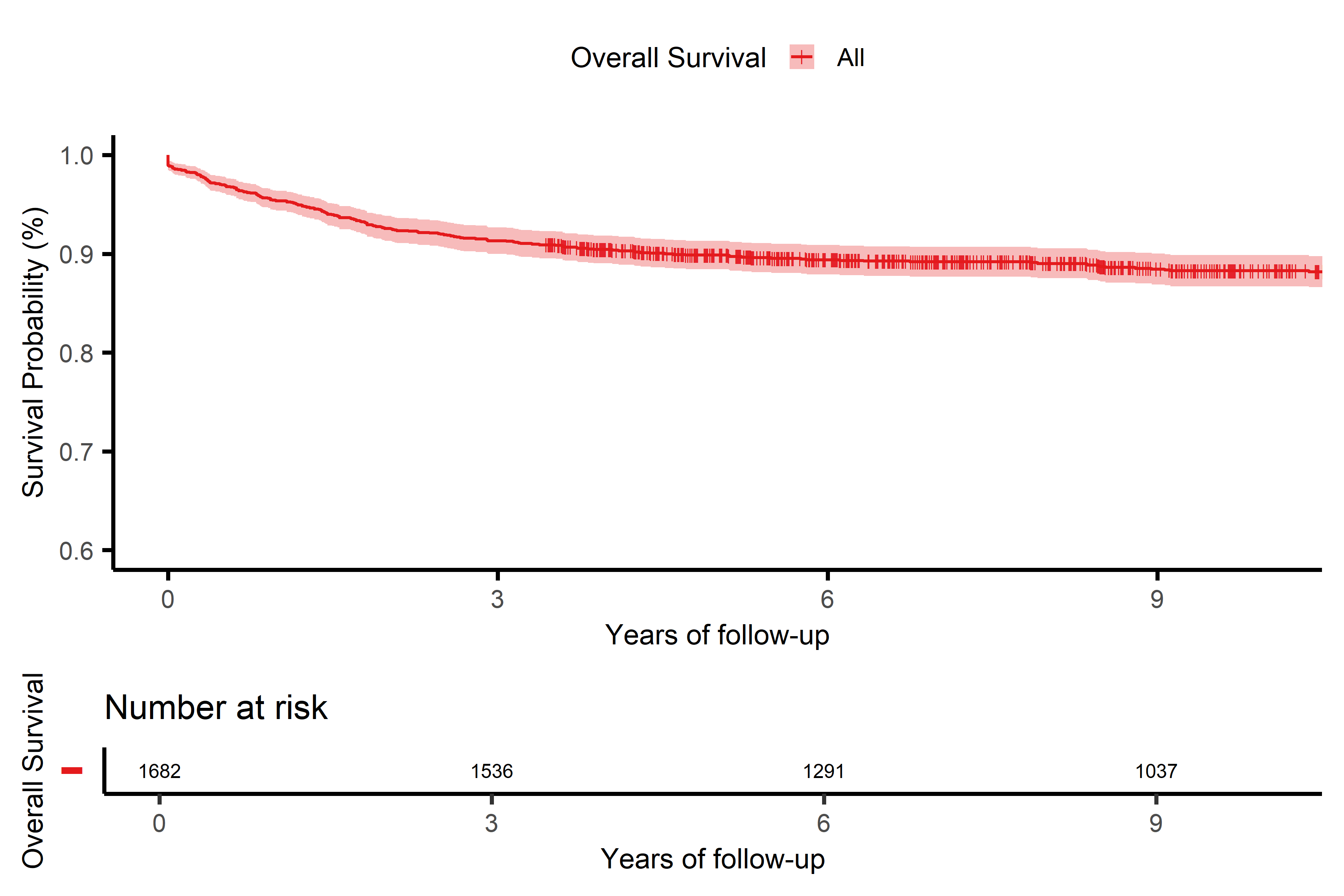
**

**Figure 1.** Survival curve for cervical cancer(CC) patients

**S1. Table 1.** Timetable of death for CC Patients

| **Time (years)** | **At Risk (n)** | **Events (n)** | **Survival Probability** | **Std. Error** | **95% CI** |
| --- | --- | --- | --- | --- | --- |
| 0 | 1682 | 18 | 0.989 | 0.0025 | 0.984; 0.994 |
| 1 | 1604 | 60 | 0.954 | 0.0051 | 0.944; 0.964 |
| 2 | 1557 | 47 | 0.926 | 0.0064 | 0.913; 0.938 |
| 3 | 1536 | 21 | 0.913 | 0.0069 | 0.900; 0.927 |
| 4 | 1467 | 15 | 0.904 | 0.0072 | 0.890; 0.918 |
| 5 | 1377 | 9 | 0.899 | 0.0074 | 0.884; 0.913 |
| 6 | 1291 | 7 | 0.894 | 0.0076 | 0.879; 0.909 |
| 7 | 1202 | 3 | 0.892 | 0.0076 | 0.877; 0.907 |
| 8 | 1117 | 2 | 0.890 | 0.0077 | 0.875; 0.905 |
| 9 | 1037 | 7 | 0.884 | 0.0079 | 0.869; 0.900 |
| 10 | 973 | 2 | 0.883 | 0.0080 | 0.867; 0.899 |
| 11 | 907 | 2 | 0.881 | 0.0081 | 0.865; 0.897 |
| 12 | 851 | 2 | 0.879 | 0.0082 | 0.863; 0.895 |
| 13 | 752 | 2 | 0.877 | 0.0083 | 0.860; 0.893 |
| 14 | 691 | 0 | 0.877 | 0.0083 | 0.860; 0.893 |
| 15 | 632 | 2 | 0.874 | 0.0085 | 0.858; 0.891 |
| 16 | 550 | 0 | 0.874 | 0.0085 | 0.858; 0.891 |
| 17 | 442 | 1 | 0.872 | 0.0087 | 0.855; 0.889 |
| 18 | 338 | 0 | 0.872 | 0.0087 | 0.855; 0.889 |
| 19 | 235 | 0 | 0.872 | 0.0087 | 0.855; 0.889 |
| 20 | 119 | 0 | 0.872 | 0.0087 | 0.855; 0.889 |
| 21 | 26 | 0 | 0.872 | 0.0087 | 0.855; 0.889 |

**S1. Figure 2**


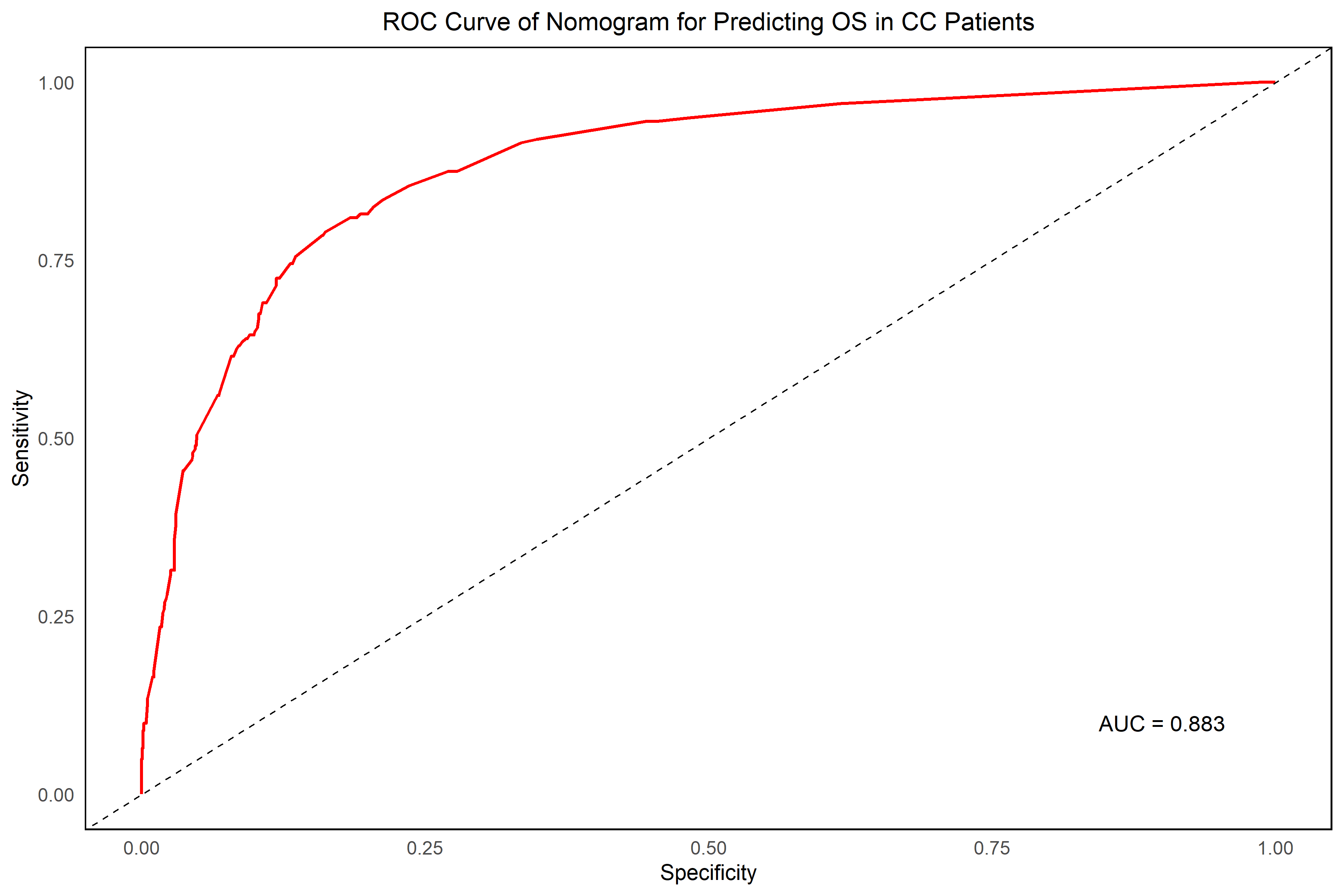


**Figure 2.** ROC Curve of the Nomogram for Predicting Overall Survival (OS) in CC Patients. The AUC is 0.881, indicating an excellent discriminative ability of the model to predict overall survival in cervical cancer patients.

**S1. Table 2.** Multicollinearity analysis of the variables in the Cox Model

| **Variables** | **GVIF** | **DF** | **GVIF^(1/(2*DF))** |
| --- | --- | --- | --- |
| Age | 1.070593 | 3 | 1.011434 |
| Skin color (Race) | 1.066783 | 2 | 1.016293 |
| City of residence | 1.056695 | 4 | 1.006917 |
| Histopathology type | 1.191537 | 3 | 1.029638 |
| Stage | 1.207645 | 2 | 1.048298 |

**S1. Figure 3:** Correlation heat map between predictor variables

**
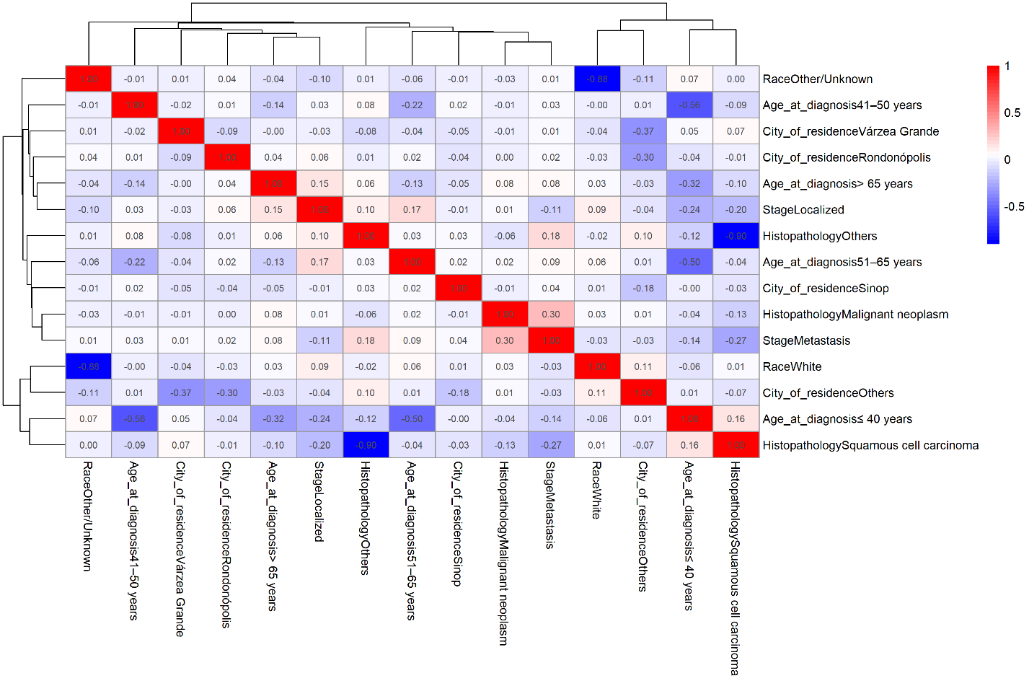
**

**Figure 3:** Heatmap of the correlation matrix among predictor variables included in the Cox regression analysis.

**S1. Figure 4:** Schoenfeld Residuals Analysis

**
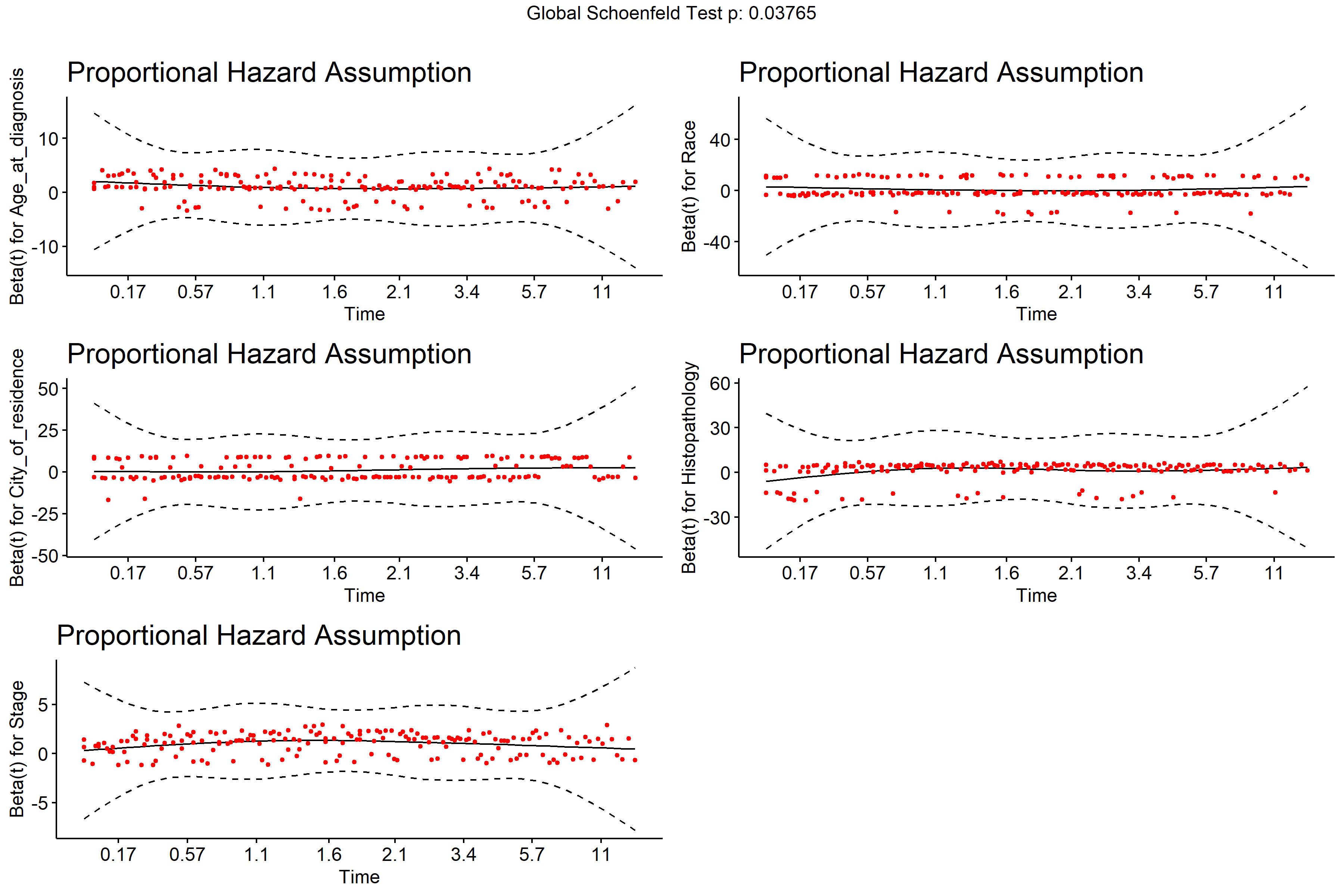
**
